# MVL-PLA2, a Snake Venom Phospholipase A2, Inhibits Angiogenesis through an Increase in Microtubule Dynamics and Disorganization of Focal Adhesions

**DOI:** 10.1371/journal.pone.0010124

**Published:** 2010-04-12

**Authors:** Amine Bazaa, Eddy Pasquier, Céline Defilles, Ines Limam, Raoudha Kessentini-Zouari, Olfa Kallech-Ziri, Assou El Battari, Diane Braguer, Mohamed El Ayeb, Naziha Marrakchi, José Luis

**Affiliations:** 1 Laboratoire des Venins et Toxines, Institut Pasteur de Tunis, Tunis, Tunisia; 2 INSERM UMR 911, CRO2, Aix-Marseille Université, Marseille, France; 3 Faculté de Médecine de Tunis, Tunis, Tunisia; Institut Pasteur, France

## Abstract

Integrins are essential protagonists of the complex multi-step process of angiogenesis that has now become a major target for the development of anticancer therapies. We recently reported and characterized that MVL-PLA2, a novel phospholipase A2 from *Macrovipera lebetina* venom, exhibited anti-integrin activity. In this study, we show that MVL-PLA2 also displays potent anti-angiogenic properties. This phospholipase A2 inhibited adhesion and migration of human microvascular-endothelial cells (HMEC-1) in a dose-dependent manner without being cytotoxic. Using Matrigel™ and chick chorioallantoic membrane assays, we demonstrated that MVL-PLA2, as well as its catalytically inactivated form, significantly inhibited angiogenesis both *in vitro* and *in vivo*. We have also found that the actin cytoskeleton and the distribution of αvβ3 integrin, a critical regulator of angiogenesis and a major component of focal adhesions, were disturbed after MVL-PLA2 treatment. In order to further investigate the mechanism of action of this protein on endothelial cells, we analyzed the dynamic instability behavior of microtubules in living endothelial cells. Interestingly, we showed that MVL-PLA2 significantly increased microtubule dynamicity in HMEC-1 cells by 40%. We propose that the enhancement of microtubule dynamics may explain the alterations in the formation of focal adhesions, leading to inhibition of cell adhesion and migration.

## Introduction

It is now well established that angiogenesis plays a critical role in tumor growth and spread [1], [2]. Tumor angiogenesis is a multistep process characterized by the chemotactic and mitogenic response of vascular endothelial cells to angiogenic growth factors, proteolytic degradation of extracellular matrix (ECM) and modulation of endothelial cell interactions with ECM components. Each of these steps may represent a potential target for the development of anti-angiogenic and anti-metastatic therapies [3]. During the angiogenic process, vascular cells become activated, resulting in the acquisition of an invasive phenotype. This invasive phenotype facilitates new vessel formation by promoting loosening of cell-cell interactions, migration and invasion of the local interstitium. Endothelial cell interactions with remodeled interstitial components then lead to morphogenesis and cellular reorganization into capillary structures. Several studies have suggested crucial roles for integrin signaling in the regulation of capillary tube formation, thus, expanding the mechanisms by which integrins regulate angiogenesis (for review see [3]).

Integrins are a family of heterodimeric transmembrane receptors that mediate cell-cell and cell-ECM interactions. These cell adhesion molecules are composed by the non-covalent association of α and β subunits. Although 18 α and 8 β subunits have been described, only 24 different combinations have been identified to date (reviewed in [4], [5]). Interactions between integrins and their ligands have been linked to many cellular processes including adhesion, migration, proliferation, differentiation, apoptosis and angiogenesis. An array of integrins has been particularly implicated in the control of angiogenesis including α1β1, α2β1, α3β1, α4β1, α5β1, α6β4, αvβ3 and αvβ5 [3], [6]. Given the importance of integrin signaling in the regulation of angiogenesis, indirect targeting of integrin function may represent a clinically useful approach for controlling aberrant neovascularization such as tumor angiogenesis. The study of the role played by integrins during angiogenesis has been mainly focused on αvβ3. This integrin interacts with several ECM proteins, such as vitronectin, fibrinogen, fibronectin, thrombin, thrombospondin and von Willebrand factor, and cooperates with molecules endowed with different biological functions, including metalloproteases, growth factors and their receptors. Due to its numerous functions and to its relatively limited cellular distribution, αvβ3 integrin represents an attractive target for therapeutic intervention [6].

Migration can be viewed as a periodically repeating sequence of events that includes formation of pseudopodial protrusions at the cell front, attachment and translocation of the cell body. Cell migration thus depends on the continuous coordinated formation and disassembly of actin-based adhesions (for review see [7]). The assembly of new focal adhesions at the leading edge is necessary for the formation of the lamellipodium and the initiation of locomotion. This assembly is due to the clustering of integrins upon their engagement with ECM components and to the subsequent recruitment of scaffolding and signaling proteins [8]. Adhesion complexes thus mechanically link the ECM to the cytoskeleton, but a concerted interaction of microtubules (MTs) and actin is essential for maintaining cell motility [9], [10]. Dynamic instability of MTs is thus required to keep the polarized actin cytoskeleton-based protrusions in the cell-leading edge [8]. Moreover, MTs promote the release of adhesion substrates by targeting adhesion complexes at the cell front and rear with different frequencies [11].

Snake venom is a natural source for molecules known as modulators of integrin-mediated functions. These proteins belong to two families, disintegrins and C-type lectin proteins (CLPs). Since their initial characterization, snake venom disintegrins have been extensively studied. They are potent and specific antagonists of several integrins, such as αvβ3 and α5β1, and can thus act on many biological processes including platelet aggregation, angiogenesis, tumor invasion and bone destruction [12], [13], [14], [15]. On the other hand, CLPs were first described as modulators of platelet aggregation before their anti-adhesive activity was highlighted. CLPs are thus able to inhibit integrin-dependent proliferation, migration, invasion and angiogenesis [16], [17], [18].

Snake venom type A2 phospholipases (PLA2s) (EC 3.1.1.4) are quite fascinating from both a biological and structural point of view. Despite their structure being conserved, they exhibit a wide range of pharmacological activities [19]. In a previous work, we showed for the first time that snake venom PLA2s inhibit adhesion and migration of tumor cells [20], [21]. We also demonstrated that these effects were not mediated by the phospholipase catalytic activity of MVL-PLA2 but through the inhibition of α5β1 and αv integrins [20]. However, to our knowledge, nothing is yet known about the potential effects of snake venom PLA2 on angiogenesis.

In the present study, we investigated the impact of integrin inhibition by MVL-PLA2, isolated from *Macrovipera lebetina* venom, on vascular endothelial cells behavior. Our results reveal that MVL-PLA2 inhibits endothelial cell adhesion and migration and abolishes angiogenesis both *in vitro* and *in vivo*. Interestingly, we also show that MVL-PLA2 enhances microtubule dynamics likely leading to the alterations in the formation of focal adhesions, which may explain its effect on angiogenesis.

## Materials and Methods

### MVL-PLA2 purification and enzymatic inactivation

The MVL-PLA2 was purified and its phospholipase catalytic activity was inactivated by *p*-bromophenacyl bromide (BPB) alkylation as described in a previous work [20].

### Cell culture

HMEC-1 cells [22] were routinely maintained at 37°C and 5% CO_2_ in MCDB-131 medium (Lonza, Levallois-Perret, France) containing 10% heat-inactivated fetal bovine serum, 2 mmol/l glutamine, 1% penicillin and streptomycin (all from Life Technologies, Paisley, UK), 1 µg/ml hydrocortisone (Pharmacia & Upjohn, St-Quentin-Yvelines, France) and 10 ng/ml epithelial growth factor (R&D Systems, Minneapolis, MN). HMEC-1 cells were grown on 0.1% gelatin-coated flasks and were used between passages 3 and 12.

### Cell adhesion and migration assays

Adhesion assays were performed as previously described [23]. Briefly, 96-well plates were coated with purified ECM protein solutions or with MVL-PLA2 for 2 h at 37°C and blocked with 0.5% PBS/BSA. Cells in single cell suspension were added to wells and allowed to adhere to the substrata for 1 h at 37°C. After washing, adherent cells were fixed with 1% glutaraldehyde, stained with 0.1% crystal violet and lysed with 1% SDS. Absorbance was then measured at 600 nm. For adhesion assay on antibodies, 96-well plates were coated with 50 µl of rabbit anti-rat IgG (50 µg/ml), overnight at 4°C. Wells were washed once with PBS and 50 µl of anti-integrin blocking antibodies (10 µg/ml) were added for 5 h at 37°C. Then, wells were blocked with 0.5% PBS/BSA and adhesion assay was continued as above.

*In vitro* cell migration assays were performed in modified Boyden chambers (NeuroProbe Inc., Bethesda, MD) with porous membranes pre-coated with 10 µg/ml of fibronectin or 50 µg/ml fibrinogen for 5 h at 37°C as previously described [18].

### *In vitro* capillary network formation on Matrigel™

250 µl of 5.25 mg/ml Matrigel™ (BD Biosciences, Pont de Claix, France) were added to 24-well plates and allowed to solidify for 1 h at 37°C. HMEC-1 cells were harvested, added to each well and incubated for 5 h at 37°C and 5% CO_2_. Cells were fixed with 4% formaldehyde and photographed using a DM-IRBE microscope (Leica, Rueil-Malmaison, France) coupled with a digital camera (CCD camera coolsnap FX, Princeton Instruments, Trenton, NJ). The formation of capillary networks was quantitatively evaluated by measuring the total capillary tube length in 20 view fields per well using Metaview software as previously described [24].

### Cytotoxicity assay

The cytotoxicity of MVL-PLA2 was determined by measuring the release of lactate dehydrogenase (LDH) activity into the medium. Suspended HMEC-1 cells were preincubated for 30 min at room temperature with MVL-PLA2 in DMEM containing 0.2% BSA and then added to Matrigel™ in 96-well plates (100 µl/well) for 5 h at 37°C. Total release of LDH (100% toxicity) was obtained by adding 0.1% Triton-X100 in incubation medium. The supernatants were collected, clarified by centrifugation 5 min at 600 g and 80 µl were submitted to LDH-based cytotoxicity kit (Sigma).

### Chick chorioallantoic membrane (CAM) angiogenesis assay

The assay was performed as previously described [13], [16]. In brief, the CAMs were prepared using 8-day-old chick embryos. Filter disks (Ø 6 mm) were soaked in 0.9% NaCl alone (control) or containing various concentrations of MVL-PLA2. After 48 h incubation, the discs were removed and the CAMs were photographed with a digital camera at ×10 magnification. To quantify CAM angiogenesis, we counted the number of vessel branching points per photographic field of the treated area using ImageJ software.

### Transfection of HMEC-1 with GFP-tagged α tubulin plasmid

The transfection of HMEC-1 was done as previously described [22]. In brief, 8×10^5^ HMEC-1 cells were transfected with 8 µg plasmid pEGFP-Tub (Clontech, Palo Alto, CA) encoding a fusion protein consisting of the human codon-optimized variant of green fluorescent protein (GFP) and the human α-tubulin gene, using transfection buffer solution R and program T-16 of a Nucleofector® (Amaxa, Cologne, Germany). DNA quantity, cell concentration, and buffer volume were kept constant throughout all experiments. After transfection, cells were immediately transferred into RPMI medium (Life Technologies) containing 10% heat-inactivated fetal bovine serum, 2 mmol/l glutamine, 1% penicillin and streptomycin, 1 µg/ml hydrocortisone, and 10 ng/ml epithelial growth factor. Cells were seeded in six-well plates onto slides pre-coated with 10 µg/ml fibronectin. Twenty-four hours later, cells were incubated for 1 h in the presence or absence of MVL-PLA2 or BPB-alkylated MVL-PLA2 and MT dynamics measurements were performed.

### Time-lapse microscopy and analysis of microtubule dynamic instability

Measurement of MT dynamic instability in living HMEC-1 cells was performed as previously described [22]. Briefly, transfected HMEC-1 cells were placed in RPMI culture medium lacking sodium bicarbonate and supplemented with 25 mmol/L HEPES, 4.5 g/l glucose and 3% (v/v) Oxyrase (Oxyrase, Inc., Mansfield, OH) to reduce photodamage. They were then placed in a double coverslip chamber maintained at 37±1°C and observed using the ×100 objective lens of an inverted fluorescence microscope (Leica). Thirty-one images per cell were acquired at 4-second intervals (see Video S1 and Video S2) using a digital camera driven by Metamorph software (Universal Imaging Corporation, Downingtown, PA) as previously described [22].

Analysis of MTs dynamics was done as described previously using the track point function of the Metamorph software [25]. Changes in length ≥0.5 µm were considered as growth or shortening events whereas changes in length <0.5 µm were considered as phases of attenuated dynamics or pauses. The rates of growth and shortening events were determined by linear regression. Means and SE were calculated per event. The catastrophe frequency based on time was calculated by dividing the number of transitions from growth or pause to shortening by the total time growing and paused for each individual MT. The rescue frequency based on time was inversely calculated, dividing the total number of transitions from shortening to pause or growth by the time spent shortening for each individual MT. Means and SE of transition frequencies were calculated per MT (n>30, for each experimental condition from three independent experiments). Dynamicity is the total length grown and shortened divided by the life span of MT population.

### Fluorescence staining

Cells were grown on slides coated with 10 µg/ml fibronectin and incubated for 48 h. Cells were incubated for 1 h in the absence (control cells) or presence of MVL-PLA2 (100 nM), fixed with formaldehyde 3.7% (15 minutes) and permeabilized with 0.1% saponin (30 minutes). Actin network was stained using TRITC-conjugated phalloidin (1/4000). Integrin αvβ3 was stained using LM609 antibody (1:500, Chemicon) and Alexa fluor-488 conjugated secondary antibody (1:2000).

### Statistical Analysis

All values are expressed as mean ± standard deviation (SD). The statistical significance of differential finding between experimental and control groups was determined by Student's t test using GraphPad Prism 4 software. P<0.05 was considered statistically significant and is indicated with asterisks over the value (*: p<0.05; **: p<0.01; ***: p<0.001).

## Results

### MVL-PLA2 inhibits endothelial cell adhesion and migration by blocking the adhesive function of α5β1 and αvβ3 integrins

We recently purified and characterized a novel snake venom PLA2, named MVL-PLA2 that impairs cell adhesion mediated by α5β1 and αv integrins in human cancer cell lines [20]. Given the key role of α5β1 and αvβ3 in angiogenesis [3], [26], [27], we decided to further evaluate the capacity of MVL-PLA2 to exert an anti-angiogenic activity. First, we investigated the cytotoxicity of MVL-PLA2 towards the human microvascular endothelial cell line-1 (HMEC-1). We have previously demonstrated that MVL-PLA2 was not cytotoxic toward human fibrosarcoma (HT1080) and melanoma (IGR39) cell lines [20]. Using the MTT assay, we found in the present study that MVL-PLA2 did not either significantly affect the viability of HMEC-1 cells when incubated for 3 days with protein concentrations up to 250 nM. We only noted a slight decrease in the number of living cells for higher concentrations (supplementary Fig. S1A), which could be due to the inhibition of cell growth. Whatever the concentration tested, neither did MVLPLA2 induce membrane damage after short term times (mimicking the pre-incubation step (30 min. in suspension), as visualized by the release of LDH (supplementary Fig. S1B).

**Figure 1:**
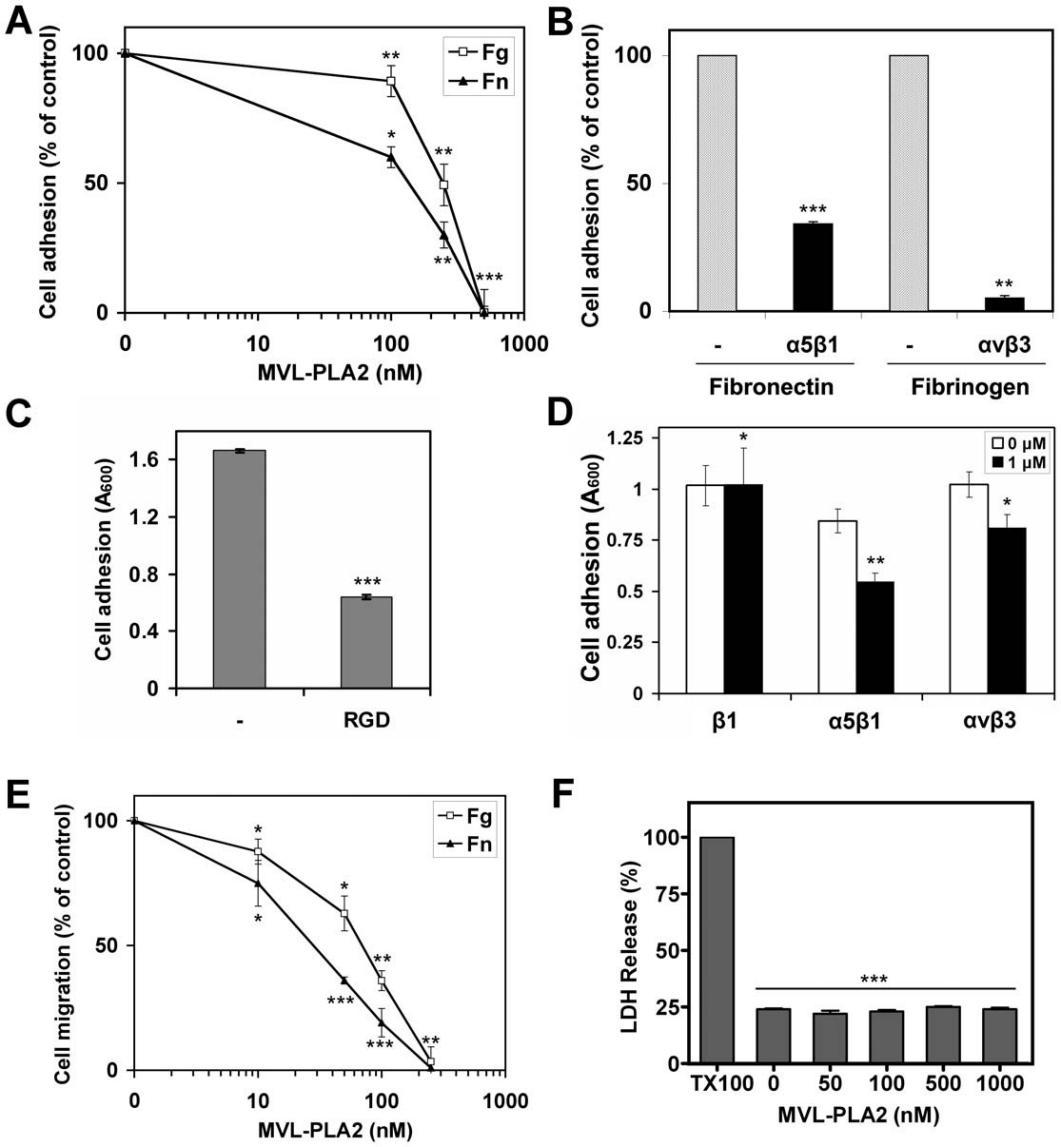
MVL-PLA2 inhibits endothelial cell adhesion and migration through α5β1 and αvβ3 integrins. (**A**) HMEC-1 cells were pre-incubated with various concentrations of MVL-PLA2 for 30 min at room temperature. Cells were then added along with the enzyme to 96-well microtiter plates coated with 10 µg/ml fibronectin (Fn, closed triangles) or 50 µg/ml fibrinogen (Fg, open squares) and allowed to adhere for 1 h at 37°C. After washing, adherent cells were stained with crystal violet, solubilized by SDS and absorbance was measured at 600 nm. Data shown are means (±SD) of at least three independent experiments and expressed as a percentage of adhesion in the absence of PLA2. (**B**) Adhesion of HMEC-1 cells, treated for 30 min at room temperature with 10 µg/ml antibodies against α5β1 (JBS5) or αvβ3 (LM609) integrins, was evaluated on fibronectin or fibrinogen, respectively. Adhesion was measured as described above. Data shown are means (±SD) of at least three independent experiments and expressed as a percentage of adhesion in the absence of antibody. (**C**) HMEC-1 cells, incubated without (−) or with 1 mM GRGDSP peptide (RGD), were tested for adhesion on microtiter plates coated with 1 µM MVL-PLA2. (**D**) HMEC-1 cells were treated without (open bars) or with (filled bars) 1 µM MVL-PLA2 and tested for adhesion on microtiter plates coated with antibodies raised against the indicated integrin as described in the experimental section. (**E**) HMEC-1 cells, treated with various concentrations of MVL-PLA2, were allowed to migrate for 5 h to fibronectin (Fn, closed triangles) or fibrinogen (Fg, open squares). Data shown (±S.D.) are expressed as a percentage of migration in the absence of peptide. (**F**) Suspended HMEC-1 cells (0.5×10^6^ cells/ml) were preincubated for 30 min at room temperature with various concentrations of MVL-PLA2 and then added along with the enzyme to Matrigel™ in 96-well plates (100 µl/well). Upon 5 h at 37°C, 80 µl of clarified supernatant were submitted to LDH activity released by damaged cells using a colorimetric assay. Total release of LDH (100% toxicity) was obtained by adding 0.1% Triton-X100 in the assay medium (TX100). Data shown (±S.D.) are from a representative experiment of three performed in triplicate.

In order to investigate whether MVL-PLA2 could inhibit endothelial cell adhesion by impairing cell/ECM interactions through an integrin-dependent mechanism, we measured the effect of various concentrations of MVL-PLA2 against HMEC-1 cell adhesion to fibronectin (Fn) and fibrinogen (Fg). As shown in Fig. 1A, MVL-PLA2 inhibited the adhesion of HMEC-1 cells to both matrices. This inhibition was dose-dependent, with half-maximal inhibition at a concentration (IC_50_) of about 170 nM and 250 nM for Fn and Fg, respectively (Fig. 1A). To determine which integrins are involved in the attachment of endothelial cells to Fn and Fg, we performed adhesion assays using function-blocking anti-α5β1 and anti-αvβ3 antibodies. As shown in Fig. 1B, anti-α5β1 antibody decreased HMEC-1 cells adhesion to Fn by 70% and anti-αvβ3 decreased adhesion to Fg by 95%. Since α5β1 and αvβ3 are both Arg-Gly-Asn (RGD)-dependent integrins, we tested the ability of HMEC-1 cells to adhere onto immobilized MVL-PLA2 in the presence of RGD peptides. As shown in Fig. 1C, we observed that the adhesion of HMEC-1 cells treated with RGD peptide was decreased by about 65% compared to untreated HMEC-1 cells. Altogether these results above suggest that MVL-PLA2 could interact with α5β1 and αvβ3 integrins in endothelial cells. To address this issue, HMEC-1 cells were treated with MVL-PLA2 for 30 min and allowed to adhere on various anti-integrin antibodies. At 1 µM, MVL-PLA2 did not affect HMEC-1 cell adhesion on anti-β1 integrin antibody (Fig. 1D). However, MVL-PLA2 reduced HMEC-1 adhesion by 35% and 21% on anti-α5β1 and anti-αvβ3 integrins antibodies, respectively. It is thus likely that the PLA2 inhibits the adhesive functions of α5β1 and αvβ3 integrins in vascular endothelial cells as previously reported for tumor cells [20].

Cell migration can be considered as a finely regulated process including successive steps of cell adhesion and de-adhesion. Because MVL-PLA2 inhibits HMEC-1 cell adhesion, we also examined its effect on cell migration using a haptotaxis assay in modified Boyden chambers. MVL-PLA2 at 250 nM completely blocked HMEC-1 cell migration towards Fn or Fg (Fig. 1E). This inhibition was dose-dependent with IC_50_ of 30 nM and 70 nM for Fn and Fg, respectively. To know whether the treatment above could affect cell integrity, suspended HMEC-1 cells were incubated with various concentrations of MVL-PLA2 for 30 min. at room temperature and then plated on Matrigel™ in the presence of the enzyme for 5h at 37°C. As illustrated in Fig. 1F, the plasma membrane integrity, visualized by LDH release, was not affected by incubation in the presence of MVL-PLA2 up to 1 µM.

### MVL-PLA2 inhibits angiogenesis *in vitro* and *in vivo*

**Figure 2:**
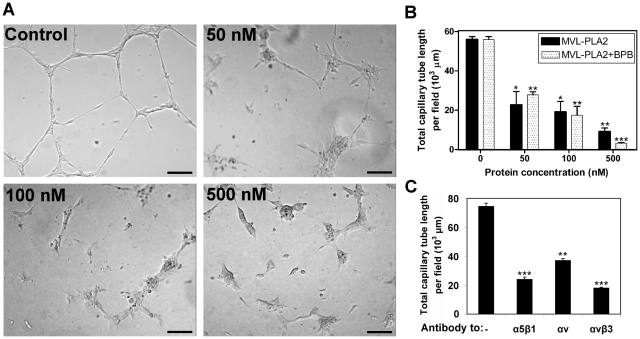
MVL-PLA2 blocks *in vitro* angiogenesis. (**A**) Representative visualization of tubulogenesis assay after pre-treatment of HMEC-1 cells without (control) or with 50, 100 or 500 nM MVL-PLA2 for 30 min at room temperature. Cells were then added to Matrigel™ in the presence of the enzyme and allowed to form capillary-like structures for 5 h at 37°C. Scale bar: 70 µm. (**B**) Dose-effect of native MVL-PLA2 or catalytically inactive MVL-PLA2 (MVL-PLA2+BPB), at the indicated concentration, on tubulogenesis on Matrigel™. Quantification of tubulogenesis was done as described in experimental section. Data shown (±S.D.) are from at least 3 independent experiments. (**C**) Tubulogenesis on Matrigel™ was performed as above in the presence of the indicated anti-integrin antibodies (10 µg/ml). Data shown (±SD) are from 3 independent experiments.

Cell adhesion and migration play a key role in the angiogenic process. Because the formation of capillary-like structures on Matrigel™ by HMEC-1 cells can be inhibited by specific anti-α5β1 and anti-αvβ3 antibodies (Fig. 2C), we hypothesized that PLA2-mediated impairment of HMEC-1 adhesion and migration could also affect endothelial cell morphogenesis. *In vitro* angiogenesis assays were thus performed using HMEC-1 cells treated with various concentrations of native MVL-PLA2 or with MVL-PLA2 catalytically inactivated by incubation with *p*-bromophenacyl bromide (BPB). As illustrated in Fig. 2A and 2B, the native MVL-PLA2 strongly inhibited *in vitro* angiogenesis in a dose-dependent manner. The inhibitory effect was observed for concentrations as low as 50 nM of peptide. In addition, we did not observe any difference in angiogenesis inhibition when the phospholipase activity of MVL-PLA2 was inactivated (Fig. 2B).

**Figure 3:**
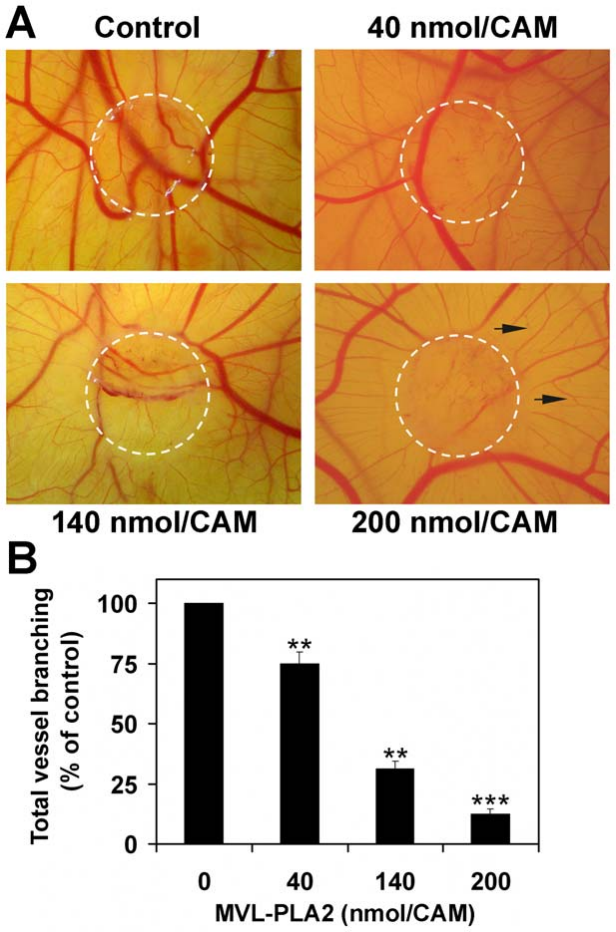
MVL-PLA2 inhibits *in vivo* angiogenesis. (**A**) Chicken CAMs were untreated (control) or treated with 40, 140 or 200 nM MVL-PLA2. White discontinued circles represent location of applied disks. Black arrows show peripheral microvessels grown centrifugally. Shown photographs are representative of 3 experiments performed in triplicate. (**B**) Quantitative measurement of neovascularisation was performed by counting the blood vessel branch points present within the treated area defined by the filter disc. Data are expressed as a percentage of control (in the absence of peptide) and are means of three independent experiments performed using five different CAMs.

To further confirm the potent anti-angiogenic properties of MVL-PLA2, we performed *in vivo* angiogenesis using chick chorioallantoic membrane (CAM) assays. As illustrated in Fig. 3A, MVL-PLA2 induced a marked reduction in the number of new capillaries and branching vessels in the CAM, without affecting the preexisting vessels. Furthermore, the peripheral vessels (relative to the position of the disc) grew centrifugally, avoiding the treated area, where a decrease in the vascular density could be observed. No sign of irritation or inflammation were observed. This effect was dose-dependent and MVL-PLA2 at 200 nM, induced a reduction of vasculature in the treated area by about 90% as compared to the control untreated CAM (Fig. 3B).

### MVL-PLA2 affects focal adhesion organization

**Figure 4:**
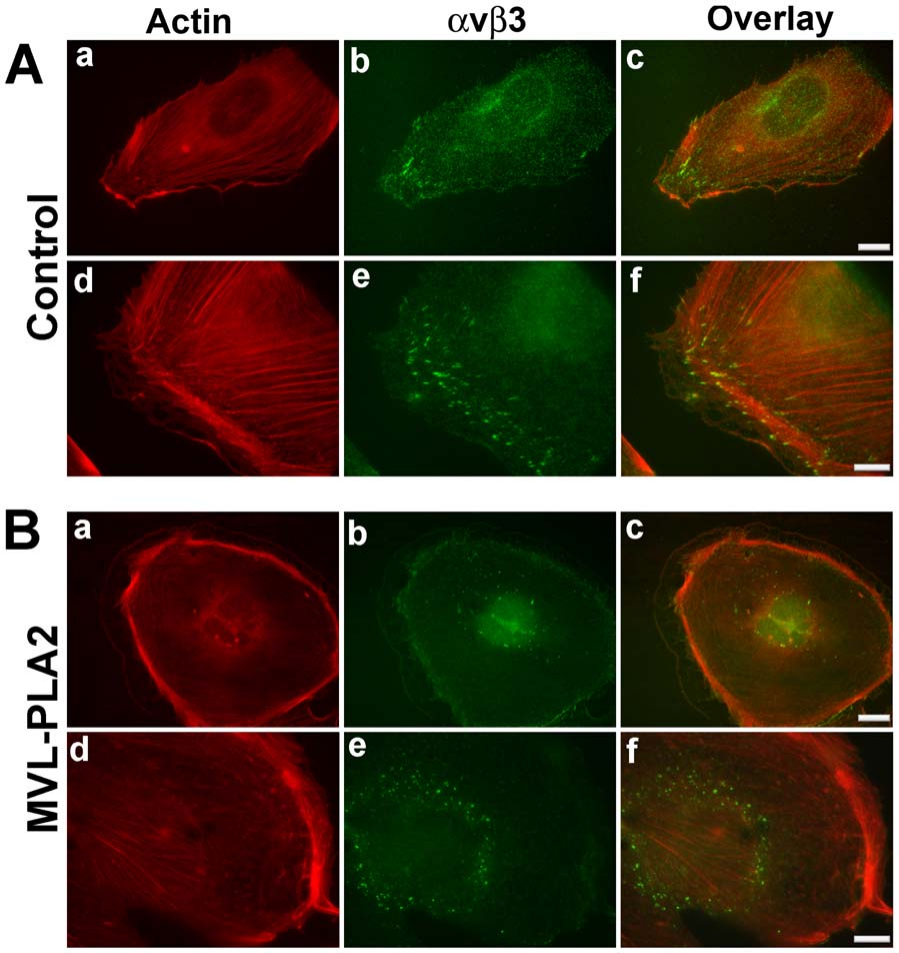
MVL-PLA2 affects focal adhesion structure. HMEC-1 cells were allowed to adhere to a fibronectin-coated surface for 48 h, treated or not (control) with 100 nM MVL-PLA2. After permeabilization, cells were co-stained for αvβ3 integrin with LM609 antibody and Alexa fluor 488-conjugated secondary antibody (*green*), and for actin with TRITC-conjugated phalloidin (*red*). Scale bars: 10 µm (a, b, c) and 4 µm (d, e, f).

Focal adhesions (FA) are specialized sites of cell attachment to the ECM where integrins receptors, such as αvβ3, link the ECM to the actin cytoskeleton, allowing traction stress to drive migration [28]. To determine whether MVL-PLA2 treatment could affect actin organization, we performed double staining of actin fibers and αvβ3 integrin. As illustrated in Fig. 4A, αvβ3 integrin (green) is concentrated in large dots over the ventral face of lamellipodia at the end of actin stress fibers (red). These dots are also stained by an antibody against paxillin, a component of FAs (see Supplemental Fig S2). Treatment of HMEC-1 cells with 100 nM MVL-PLA2 for 1 h induced important changes in cell morphology. Treated cells have a circular shape and actin stress fibers are thinner or absent, with the actin mainly located at the cell periphery (Fig. 4B). Moreover, treatment by MVL-PLA2 resulted in a drastic reduction in the size of FAs and their redistribution all over the ventral surface of cells (Supplemental Fig S2), consistent with a decrease in αvβ3 integrin clustering and its absence from lamellipodia (Fig. 4B). Therefore, it appears that the inhibition of migration by MVL-PLA2 treatment is associated with important reorganization of the actin cytoskeleton and FAs.

### MVL-PLA2 increases microtubule dynamics in living human endothelial cells

Endothelial cell migration is a complex process that requires an extensive reorganization of the cytoskeleton (for review see [8]). Recent studies have pointed out an important role of MTs and coordinated MT/actin dynamics in regulating cell migration [9], [10]. The dynamic properties of MTs, key components of the cytoskeleton, are highly regulated both spatially and temporally and this regulation is crucial for cell migration (for review see [29], [30]). Thus, we postulated that MVL-PLA2 might inhibit endothelial cell migration by affecting MT dynamics. To verify this hypothesis, we analyzed the dynamic instability behavior of MTs in living HMEC-1 after 1 h incubation with 100 nM of native or BPB-inactivated MVL-PLA2.

**Figure 5:**
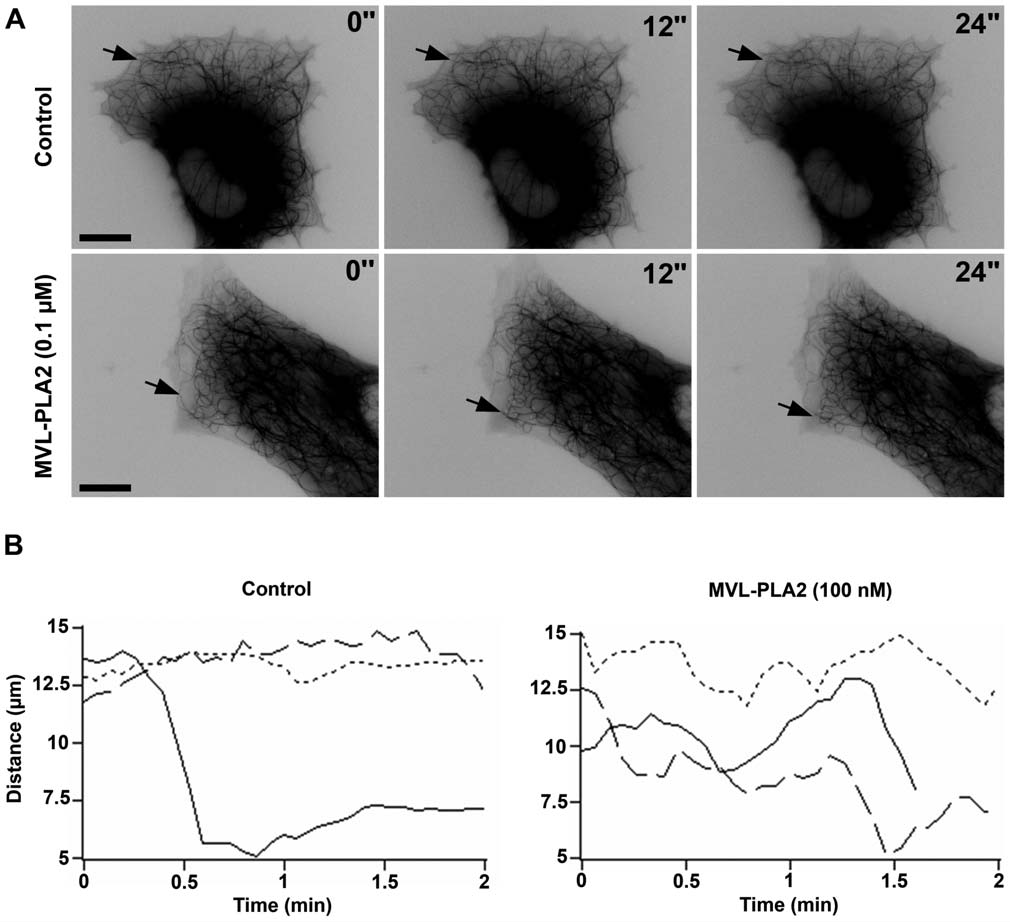
MVL-PLA2 increases microtubule dynamics in living HMEC-1. (**A**) Time-lapse sequence of video frames of the plus ends of several MTs in a control cell (top panels) or in a cell treated with 100 nM MVL-PLA2 (bottom panels). Arrowheads indicate one MT end for which the position did not change over a period of 24 s. in the control cell and one MT that underwent a shortening event in the treated cell. Time is indicated in seconds (″). Scale bar: 10 µm. (**B**) Life history plots of the length changes of 3 representative MTs in living HMEC-1 cells treated or not (control) with 100 nM of MVL-PLA2 as described in experimental procedure section. In the presence of 100 nM of MVL-PLA2, MTs are characterized by more extensive growth and shortening events compared with control cells.

Fig. 5A shows an image gallery of the lamellar region of a control untreated HMEC-1 cell (top panels) and a HMEC-1 cell incubated with 100 nM native MVL-PLA2 (bottom panels). In the control cells, the MT plus ends alternated between phases of slow growing, rapid shortening and prolonged pause state (a state of attenuated dynamic instability). Interestingly, 100 nM of MVL-PLA2 markedly increased MT dynamics in HMEC-1 cells (see videos S1 and S2 in supplementary data). For example, the plus end of the MT indicated by arrowheads in the bottom panel of Fig. 5A shortened for a long distance in a short period of time (12 seconds). Fig. 5B shows the life history traces of the changes in length of 3 individual MTs in the absence (control) or in the presence of 100 nM MVL-PLA2. The life history traces of MTs in cells incubated with MVL-PLA2 show extensive length changes as compared to the MTs from control cells.

At least 30 individual MTs per condition were analyzed to determine the dynamic instability parameters. The effects of MVL-PLA2 on these various variables are presented in Table 1. In control cells, the overall MT dynamicity was 3.0 µm/min with a mean growth rate of 7.12±0.29 µm/min, slower than their mean shortening rate of 8.79±0.38 µm/min. The mean growing and shortening lengths were 1.06±0.03 and 1.32±0.05 µm, respectively and MTs spent 63% of their time in a paused state, neither growing nor shortening to a detectable extent. After 1 h incubation with MVL-PLA2 (100 nM), the percentage of dynamic MTs was significantly increased (from 58.5% in control cells to 78.1% in MVL-PLA2-treated cells) and the overall MT dynamicity was increased by 40%. This increase in MT dynamicity induced by MVL-PLA2 was due to an increase in both the growth and shortening rates (+31% and +13%, respectively) and a decrease in the duration of pauses (−22%), leading to a decrease in the percentage of time spent in pause (from 63.1% to 56.4%).

**Table 1:** Effects of MVL-PLA2 on the variables of microtubule dynamic instability in living HMEC-1 cells.

Variables		Control	100 nM MVL-PLA2	% change	100 nM BPB-inactivated MVL-PLA2	% change
**% of dynamic microtubules**		58.5±4.0%	78.1±4.1%	**	76.7±7.4%	*
**Rate (µm/min)**	**G**	7.12±0.29	9.35±0.36	+31% ***	7.71±0.50	NS
	**S**	8.79±0.38	9.92±0.45	+13% **	12.54±0.83	+43% ***
**Length (µm)**	**G**	1.06±0.03	1.03±0.04	NS	1.34±0.07	+26% ***
	**S**	1.32±0.05	1.29±0.06	NS	1.81±0.13	+37% ***
**Duration (min)**	**G**	0.149±0.008	0.110±0.004	−26% ***	0.174±0.012	NS
	**S**	0.150±0.006	0.130±0.006	−13% *	0.145±0.008	NS
	**P**	0.315±0.015	0.247±0.013	−22% ***	0.238±0.019	−24% **
**% time spent**	**G S P**	15.4 21.5 63.1	18.7 24.9 56.4		27 19.5 53.4	
**Transition frequency (min^−1^)**	**C**	2.25±0.26	2.63±0.17	NS	2.15±0.30	NS
	**R**	6.48±0.35	7.75±0.42	+20% *	6.48±0.62	NS
**Transition frequency (µm^-1^)**	**C**	1.74±0.16	1.48±0.14	NS	1.18±0.21	−32% *
	**R**	0.88±0.04	1.01±0.05	NS	0.64±0.08	−27% **
**Dynamicity (µm/min)**		**3.0**	**4.2**	**+40%**	**4.5**	**+50%**

Variables of microtubule dynamic instability were determined on living HMEC-1 cells after 1 h incubation with 100 nM native or BPB-inactivated MVL-PLA2. G: growth; S: shortening; P: pause; C: catastrophe; R: rescue; NS: non significant.

Student's *t* test: * p<0.05; ** p<0.01; *** p<0.001.

In addition, as shown in Table 1, no significant difference was observed between the effect of the native form of MVL-PLA2 and its catalytically inactivated form. Indeed, incubation of HMEC-1 cells with 100 nM BPB-inactivated MVL-PLA2 resulted in a significant increase in the percentage of dynamic MTs (from 58.5% to 76.7%) and a 50% increase in overall MT dynamicity. As for the native form of MVL-PLA2, the increase in MT dynamicity induced by inactivated MVL-PLA2 was due to an increase in the shortening rate (+43%) and a decrease in the mean duration of pause (−24%), resulting in a decrease in the percentage of time spent in pause (from 63.1% to 53.4%). The main difference between the two forms of MVL-PLA2 was the lack of increase in the MT growth rate observed with the catalytically inactivated MVL-PLA2 (Table 1). These results further confirmed the dissociation between the enzymatic activity of MVL-PLA2 and its anti-angiogenic properties.

## Discussion

Angiogenesis is the formation of new capillaries from pre-existing blood vessels. Since Folkman first formulated the hypothesis that tumor growth was angiogenesis-dependent in 1971 [31], intensive research has been carried out to confirm this theory. As a result, tumor-recruited microvascular endothelial cells have become an important target in cancer therapy and a number of anti-angiogenic factors are currently being tested in clinical trials (for review see [32]). Some of these agents are known to show anti-angiogenic and anti-tumor activity *via* their ability to interact with distinct integrins expressed in angiogenic endothelial cells of tumor vasculature [3]. Numerous anti-angiogenic components targeting integrin receptors have been isolated from snake venom. Among these peptides, the disintegrin family was the first to be characterized and the most extensively studied [15]. More recently, we demonstrated that snake venom-derived C-type lectins, such as lebectin from *Macrovipera lebetina* venom, also displayed anti-angiogenic properties [16]. In the present study, we extend these findings to another snake venom family and reveal the anti-angiogenic properties of a newly discovered snake venom PLA2, MVL-PLA2. This is the first report describing this new pharmacological effect of a snake venom PLA2 and delineating its mechanism of action.

The present study was based on earlier observations showing a potent inhibitory effect of MVL-PLA2 on tumor cell adhesion and migration [20]. Our results showed that MVL-PLA2 specifically targets α5β1 and αv-containing integrins in tumor cells. Since α5β1 and αvβ3 integrin receptors are key actors of tumor angiogenesis (for review see [3], [26], [27]), we postulated that MVL-PLA2 could have an anti-angiogenic potential. Interestingly, we demonstrated here that MVL-PLA2 impairs adhesion and migration of vascular endothelial cell, as well as *in vitro* and *in vivo* angiogenesis. This anti-angiogenic activity is not likely to be due to any cytotoxic effect of MVL-PLA2 on endothelial cells. Indeed, whatever the concentration used, the venom PLA2 did not induce any plasma membrane damage, as no release of LDH was observed upon the preincubation step (see supplemental Fig S1B) or upon plating for 5 h (see Fig. 1F). Moreover, proliferation of HMEC-1 cells was not affected by MVL-PLA2 over a 3-day period for concentrations affecting angiogenesis (see supplemental Fig S1), cells did not detach from the substratum during this period and no cell debris could be observed (data not shown).

Elsewhere, by inactivating the PLA2 enzymatic site, we previously showed a clear dissociation of the anti-tumor effect of MVL-PLA2 and its catalytic activity [20]. The same observation was made in the present study for the anti-angiogenic activity of MVL-PLA2. Hence, our findings strongly support the concept of target model proposed by Kini *et al.* [19] to explain the pharmacological effects of PLA2s. According to this model, high affinity protein-protein interactions between PLA2 and membrane protein in the target cells determine the specific pharmacological effects of PLA2 enzymes.

Our results on tumor cells [20] and endothelial cells (this study) suggest that MVL-PLA2 could interfere with α5β1 and αvβ3 integrins function. Angiogenesis involves *de novo* expression of the integrin αvβ3, which binds to RGD-containing components of the interstitial matrix [33]. Furthermore, α5β1 integrin is expressed in growing vessels, but its expression disappears in mature vessels (for a review see [34]). Consistent with this, our results showed that MVL-PLA2 does not affect stable, established blood vessels on CAMs, but strongly reduces the number of new capillaries and vascular branching.

It is well established that RGD-containing peptides have anti-angiogenic properties due in part to inhibition of αv-dependent adhesion and can prevent vascular lumen formation [35], [36]. We show here that endothelial cells are able to adhere on immobilized MVL-PLA2 and that this adhesion is impaired by RGD peptides. Similar results were obtained with tumor cells [20]. This suggests that the interaction between MVL-PLA2 and integrins involves an RGD-like sequence which may be responsible for the inhibition of integrin function. MVL-PLA2 contains a NGD similar sequence that may be responsible for the inhibition of integrin function. Further structure-function relationships studies must be carried out to verify this hypothesis.

Our findings clearly show that MVL-PLA2 efficiently abolishes adhesion and migration of endothelial cells and capillary tube formation. These processes are all linked and depend on reorganization of the cytoskeleton. Directed cell migration requires that cell-generated traction stresses exerted on the ECM are spatially and temporally coordinated [37]. Traction stresses are thought to originate in the actin cytoskeleton and be transmitted to the ECM through FA [28]. We show here that MVL-PLA2 induces a reduction in the size of FAs and their redistribution all over the ventral surface of cells. MVL-PLA2 also impedes integrin αvβ3 clustering and its localization in lamellipodia. These results are in concordance with the work of Castel *et al.* [38], showing that treatment of M21 melanoma cells with αv integrin antagonists, notably RGD-peptides, disrupts the actin cytoskeleton, redistributes αv integrins and induces molecular disassembly of focal contacts.

Cell motility is the result of a complex series of events that must be integrated both spatially and temporally [39]. Efficient motility requires the coordinated regulation of actin, MTs, and adhesion sites throughout the migration process [37]. Incorporation of EGFP-tagged α tubulin into MTs allows the tracking of individual MT plus ends and the analysis of MT dynamics. Interestingly, we found that treatment with native or enzymatically inactivated MVL-PLA2, dramatically increased MT dynamicity in HMEC-1 cells. To our knowledge, this is the first report describing the effects of a secreted PLA2 on MT dynamics. Microtubule targeting agents are known to have anti-angiogenic effects through modulation of MT dynamicity (for review see [40]). MTs are essential in the orchestration of endothelial cell motility [41], [42]. It is thus likely that the specific mechanism of action of MVL-PLA2 (i.e., interference with cytoskeleton dynamics) is responsible for inhibition of the angiogenic process.

Cells treated with MVL-PLA2 presented a disorganized F-actin and lost their migrating shape. Actin stress fibers are clearly visible in migrating cells but are thinner or absent in MVL-PLA2-treated-cells. In this case, we observed the development of circumferential actin bundles instead of straight fibers (see Fig. 4B). Pletjushkina *et al.* have reported the same observation in rat fibroblasts after treatment with paclitaxel [43], suggesting that MT dynamics is required for actin polymerization. Furthermore, it has been recently reported that alteration of MT dynamics is associated with changes in actin cytoskeleton and defective FA assembly in endothelial and breast carcinoma cells [41], [42].

The mechanism of action of MVL-PLA2 on MT dynamics remains unknown. However, it has long been known that MTs control local activation of Rho GTPases, Rac and Cdc42, and that alteration of MT dynamics leads to a depolarized morphology and to an inhibition of directional migration [44]. On the other hand, it has been proposed that Rac, transiently activated by new integrin binding, selectively stabilizes MTs toward the front of the cell. Since MTs target FAs to accelerate their turnover, this could favor new adhesion formation at the leading edge of the cell [45]. Furthermore, it was recently shown that RhoA-mDia1 signaling regulates MT dynamics, FA formation, and the organization of the lamellipodial actin network [42]. Based on our data, we propose that the observed increase in MT dynamics in MVL-PLA2-treated cells could affect cell polarization and the turnover of FAs, leading to the impairment of directional migration. We are aware that this proposal remains speculative and future studies will thus be carried out to check whether MVL-PLA2 interferes with Rho GTPases signaling cascade.

We demonstrate here that MVL-PLA2 abolishes *in vitro* and *in vivo* angiogenesis through inhibition of α5β1 and αv integrins function. In addition, we report original findings on the anti-angiogenic mechanism of MVL-PLA2 and demonstrate that treatment of endothelial cells with MVL-PLA2 results in an increase in MT dynamics, a disorganization of the F-actin network and alterations in FA formation. Further studies should be carried out to completely unveil the anti-angiogenic mechanism of MVL-PLA2. Nevertheless, MVL-PLA2 could represent a new potential integrin antagonist for anti-angiogenic and anti-metastatic therapy.

## Supporting Information

Figure S1 MVL-PLA2 does not significantly affect HMEC-1 viability. (A) HMEC-1 cells, seeded in 96-well plates, were treated with various concentrations of MVL-PLA2 for 72 h. After incubation 3 hours with 0.5 mg/ml 3-(4,5-dimethylthiazol-2-yl)-2,5-diphenyltetrazolium bromide (MTT), the stain was eluted with 100 Î¼l DMSO and absorbance was measured at 550 nm. (B) Suspended HMEC-1 cells (0.5×106 cells/ml) were treated with various concentrations of MVL-PLA2 for 30 min Ã room temperature. The LDH activity released by damaged cells was measured by a colorimetric assay on 80 Âµl of clarified supernatant. Total release of LDH (100% toxicity) was obtained in the presence of 0.1% Triton-X100 in the medium (TX100).
(0.54 MB TIF)

Figure S2 MVL-PLA2 alter the size and the cell distribution of FA HMEC-1 cells were treated or not with 100 nM MVL-PLA2, permeabilized and co-stained for paxillin with anti-paxillin antibody (green) and for actin with TRITC-conjugated phalloidin (red). Scale bars: 10 Âµm.
(9.97 MB TIF)

Video S1 HMEC-1 cells expressing GFP-tubulin were treated without MVL-PLA2 for 1 h and observed using the ×100 objective lens of an inverted fluorescence microscope (Leica). Thirty-one images per cell were acquired at 4-second intervals using a digital camera driven by Metamorph software as previously described (Pourroy et al. (2006) Cancer Res 66: 3256).
(8.41 MB ZIP)

Video S2 Microtubule dynamics in MVL-PLA2-treated HMEC-1 cells. HMEC-1 cells expressing GFP-tubulin were treated with 100 nM MVL-PLA2 for 1 h and observed using the ×100 objective lens of an inverted fluorescence microscope (Leica). Thirty-one images per cell were acquired at 4-second intervals using a digital camera driven by Metamorph software as previously described (Pourroy et al. (2006) Cancer Res 66: 3256).
(8.55 MB ZIP)

## References

[1] Folkman J (1972). Anti-angiogenesis: new concept for therapy of solid tumors.. Ann Surg.

[2] Garmy-Susini B, Varner JA (2008). Roles of integrins in tumor angiogenesis and lymphangiogenesis.. Lymphat Res Biol.

[3] Akalu A, Cretu A, Brooks PC (2005). Targeting integrins for the control of tumour angiogenesis.. Expert Opin Investig Drugs.

[4] Humphries MJ (2000). Integrin structure.. Biochem Soc Trans.

[5] Hynes RO (2002). Integrins: bidirectional, allosteric signaling machines.. Cell.

[6] Alghisi GC, Ruegg C (2006). Vascular integrins in tumor angiogenesis: mediators and therapeutic targets.. Endothelium.

[7] Burridge K, Fath K, Kelly T, Nuckolls G, Turner C (1988). Focal adhesions: transmembrane junctions between the extracellular matrix and the cytoskeleton.. Annu Rev Cell Biol.

[8] Wehrle-Haller B, Imhof BA (2003). Actin, microtubules and focal adhesion dynamics during cell migration.. Int J Biochem Cell Biol.

[9] Rodriguez OC, Schaefer AW, Mandato CA, Forscher P, Bement WM (2003). Conserved microtubule-actin interactions in cell movement and morphogenesis.. Nat Cell Biol.

[10] Wu X, Kodama A, Fuchs E (2008). ACF7 Regulates Cytoskeletal-Focal Adhesion Dynamics and Migration and Has ATPase Activity.. Cell.

[11] Small JV, Kaverina I (2003). Microtubules meet substrate adhesions to arrange cell polarity.. Curr Opin Cell Biol.

[12] Andrews RK, Berndt MC (2000). Snake venom modulators of platelet adhesion receptors and their ligands.. Toxicon.

[13] Olfa KZ, Jose L, Salma D, Amine B, Najet SA (2005). Lebestatin, a disintegrin from Macrovipera venom, inhibits integrin-mediated cell adhesion, migration and angiogenesis.. Lab Invest.

[14] Calvete JJ, Marcinkiewicz C, Sanz L (2007). KTS and RTS-disintegrins: anti-angiogenic viper venom peptides specifically targeting the alpha 1 beta 1 integrin.. Curr Pharm Des.

[15] McLane MA, Joerger T, Mahmoud A (2008). Disintegrins in health and disease.. Front Biosci.

[16] Pilorget A, Conesa M, Sarray S, Michaud-Levesque J, Daoud S (2007). Lebectin, a Macrovipera lebetina venom-derived C-type lectin, inhibits angiogenesis both in vitro and in vivo.. J Cell Physiol.

[17] Sarray S, Berthet V, Calvete JJ, Secchi J, Marvaldi J (2004). Lebectin, a novel C-type lectin from Macrovipera lebetina venom, inhibits integrin-mediated adhesion, migration and invasion of human tumour cells.. Laboratory Investigation.

[18] Sarray S, Delamarre E, Marvaldi J, El Ayeb M, Marrakchi N (2007). Lebectin and lebecetin, two C-type lectins from snake venom, inhibit a5b1 and av-containing integrins.. Matrix Biol.

[19] Kini RM (2003). Excitement ahead: structure, function and mechanism of snake venom phospholipase A2 enzymes.. Toxicon.

[20] Bazaa A, Luis J, Srairi-Abid N, Kallech-Ziri O, Kessentini-Zouari R (2009). MVL-PLA2, a phospholipase A2 from Macrovipera lebetina transmediterranea venom, inhibits tumor cells adhesion and migration.. Matrix Biol.

[21] Zouari-Kessentini R, Luis J, Karray A, Kallech-Ziri O, Srairi-Abid N (2009). Two purified and characterized phospholipases A2 from Cerastes cerastes venom, that inhibit cancerous cell adhesion and migration.. Toxicon.

[22] Pourroy B, Honore S, Pasquier E, Bourgarel-Rey V, Kruczynski A (2006). Antiangiogenic concentrations of vinflunine increase the interphase microtubule dynamics and decrease the motility of endothelial cells.. Cancer Res.

[23] Delamarre E, Taboubi S, Mathieu S, Berenguer C, Rigot V (2009). Expression of integrin alpha6beta1 enhances tumorigenesis in glioma cells.. Am J Pathol.

[24] Pasquier E, Carre M, Pourroy B, Camoin L, Rebai O (2004). Antiangiogenic activity of paclitaxel is associated with its cytostatic effect, mediated by the initiation but not completion of a mitochondrial apoptotic signaling pathway.. Mol Cancer Ther.

[25] Pasquier E, Honore S, Pourroy B, Jordan MA, Lehmann M (2005). Antiangiogenic concentrations of paclitaxel induce an increase in microtubule dynamics in endothelial cells but not in cancer cells.. Cancer Res.

[26] Serini G, Valdembri D, Bussolino F (2006). Integrins and angiogenesis: a sticky business.. Exp Cell Res.

[27] Hodivala-Dilke K (2008). alphavbeta3 integrin and angiogenesis: a moody integrin in a changing environment.. Curr Opin Cell Biol.

[28] Gardel ML, Sabass B, Ji L, Danuser G, Schwarz US (2008). Traction stress in focal adhesions correlates biphasically with actin retrograde flow speed.. J cell Biol.

[29] Honoré S, Pasquier E, Braguer D (2005). Understanding microtubule dynamics for improved cancer therapy.. Cell Mol Life Sci.

[30] Pasquier E, Honoré S, Braguer D (2006). Microtubule-targeting agents in angiogenesis: where do we stand?. Drug Resist Updat.

[31] Folkman J (1971). Tumor angiogenesis: therapeutic implications.. N Engl J Med.

[32] Folkman J (2007). Angiogenesis: an organizing principle for drug discovery?. Nat Rev Drug Discov.

[33] Drake CJ, Cheresh DA, Little CD (1995). An antagonist of integrin alpha v beta 3 prevents maturation of blood vessels during embryonic neovascularization.. J Cell Sci.

[34] Stupack DG, Cheresh DA (2004). Integrins and angiogenesis.. Curr Top Dev Biol.

[35] Kim SY, Oh HK, Ha JM, Ahn HY, Shin JC (2007). RGD-peptide presents anti-adhesive effect, but not direct pro-apoptotic effect on endothelial progenitor cells.. Arch Biochem Biophys.

[36] Sheu JR, Yen MH, Kan YC, Hung WC, Chang PT (1997). Inhibition of angiogenesis in vitro and in vivo: comparison of the relative activities of triflavin, an Arg-Gly-Asp-containing peptide and anti-alpha(v)beta3 integrin monoclonal antibody.. Biochim Biophys Acta.

[37] Lauffenburger DA, Horwitz AF (1996). Cell migration: a physically integrated molecular process.. Cell.

[38] Castel S, Pagan R, Garcia R, Casaroli-Marano RP, Reina M (2000). Alpha v integrin antagonists induce the disassembly of focal contacts in melanoma cells.. Eur J Cell Biol.

[39] Ridley AJ, Schwartz MA, Burridge K, Firtel RA, Ginsberg MH (2003). Cell migration: integrating signals from front to back.. Science.

[40] Pasquier E, Andre N, Braguer D (2007). Targeting microtubules to inhibit angiogenesis and disrupt tumour vasculature: implications for cancer treatment.. Curr Cancer Drug Targets.

[41] Honoré S, Pagano A, Gauthier G, Bourgarel-Rey V, Verdier-Pinard P (2008). Antiangiogenic vinflunine affects EB1 localization and microtubule targeting to adhesion sites.. Mol Cancer Ther.

[42] Zaoui K, Honore S, Isnardon D, Braguer D, Badache A (2008). Memo-RhoA-mDia1 signaling controls microtubules, the actin network, and adhesion site formation in migrating cells.. J Cell Biol.

[43] Pletjushkina OJ, Ivanova OJ, Kaverina IN, Vasiliev JM (1994). Taxol-treated fibroblasts acquire an epithelioid shape and a circular pattern of actin bundles.. Exp Cell Res.

[44] Etienne-Manneville S (2004). Actin and microtubules in cell motility: which one is in control?. Traffic.

[45] Moissoglu K, Schwartz MA (2006). Integrin signalling in directed cell migration.. Biol Cell.

