# Supplementary figures and images for: MVL-PLA2, a Snake Venom Phospholipase A2, Inhibits Angiogenesis through an Increase in Microtubule Dynamics and Disorganization of Focal Adhesions
Source: PLoS One. 2010 Apr 12;5(4):e10124. doi: 10.1371/journal.pone.0010124 (PMC2853567; doi:10.1371/journal.pone.0010124)

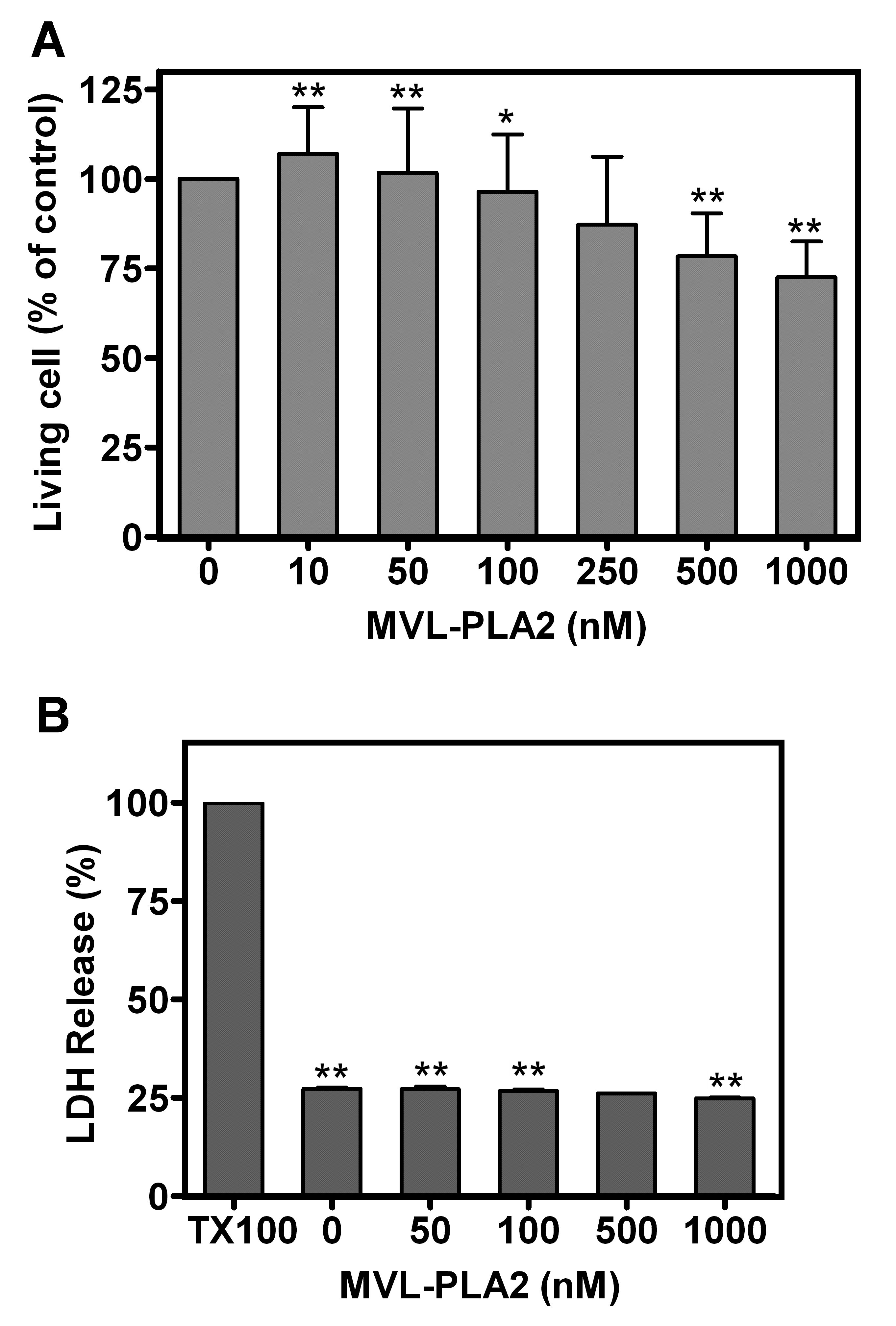

Supplement: Figure S1 — MVL-PLA2 does not significantly affect HMEC-1 viability. (A) HMEC-1 cells, seeded in 96-well plates, were treated with various concentrations of MVL-PLA2 for 72 h. After incubation 3 hours with 0.5 mg/ml 3-(4,5-dimethylthiazol-2-yl)-2,5-diphenyltetrazolium bromide (MTT), the stain was eluted with 100 Î¼l DMSO and absorbance was measured at 550 nm. (B) Suspended HMEC-1 cells (0.5×10^6^ cells/ml) were treated with various concentrations of MVL-PLA2 for 30 min Ã room temperature. The LDH activity released by damaged cells was measured by a colorimetric assay on 80 Âµl of clarified supernatant. Total release of LDH (100% toxicity) was obtained in the presence of 0.1% Triton-X100 in the medium (TX100). (0.54 MB TIF) [file pone.0010124.s001.tif]

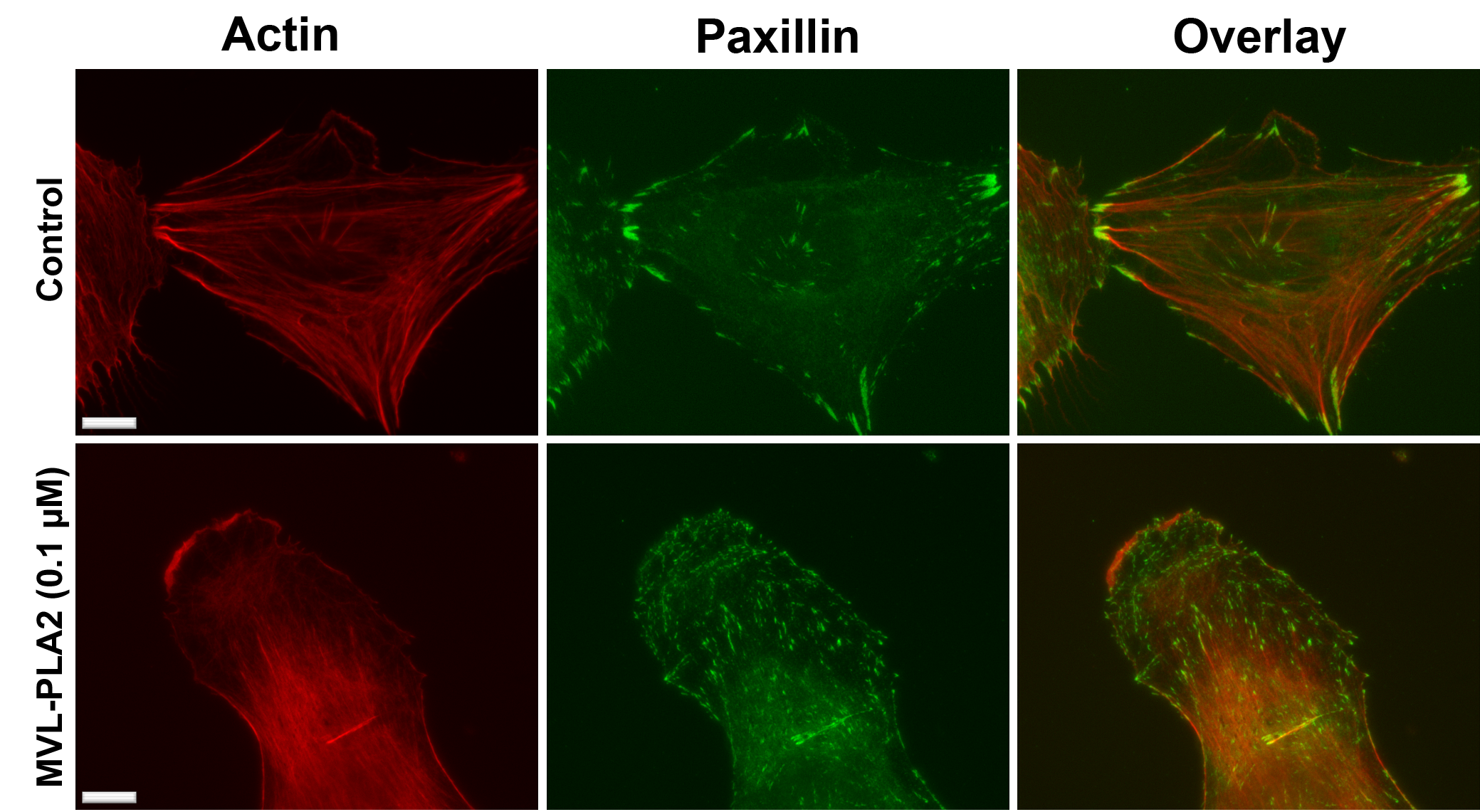

Supplement: Figure S2 — MVL-PLA2 alter the size and the cell distribution of FA HMEC-1 cells were treated or not with 100 nM MVL-PLA2, permeabilized and co-stained for paxillin with anti-paxillin antibody (green) and for actin with TRITC-conjugated phalloidin (red). Scale bars: 10 Âµm. (9.97 MB TIF) [file pone.0010124.s002.tif]
